# Enhanced Effect of Combining Chlorogenic Acid on Selenium Nanoparticles in Inhibiting Amyloid β Aggregation and Reactive Oxygen Species Formation In Vitro

**DOI:** 10.1186/s11671-018-2720-1

**Published:** 2018-09-29

**Authors:** Licong Yang, Na Wang, Guodong Zheng

**Affiliations:** Jiangxi Key Laboratory of Natural Product and Functional Food, College of Food Science and Engineering, Jiangxi Agricultural University, Nanchang, 330045 China

**Keywords:** Amyloid-β, Anti-oxidation, Anti-aggregation, Selenium nanoparticles, Chlorogenic acid

## Abstract

The deposition of amyloid-β (Aβ) plaques and formation of neurotoxic reactive oxygen species (ROS) is a significant pathological signature of Alzheimer’s disease (AD). Herein, a novel strategy is reported for combining the unique Aβ absorption property of selenium nanoparticles with the natural antioxidant agent chlorogenic acid (CGA) to form CGA@SeNPs. The in vitro biological evaluation revealed that CGA could clear the ROS induced by Aβ40 aggregates, but it did not inhibit the Aβ40 aggregation and cell membrane damage which were also caused by Aβ40 aggregates. Interestingly, CGA@SeNPs show an enhanced inhibition effect on Aβ40 aggregation and, more importantly, protect PC12 cells from Aβ aggregation-induced cell death. It is believed that CGA@SeNPs are more efficient than CGA in reducing Aβ40 toxic in long-term use.

**Electronic supplementary material:**

The online version of this article (10.1186/s11671-018-2720-1) contains supplementary material, which is available to authorized users.

## Background

Alzheimer’s disease (AD) is an irreversible progressive neurodegenerative disease characterized by progressive cognitive impairments and neuronal loss [1]. It is now widely accepted that the accumulation of extracellular amyloidal plaques in the brain is a common pathological feature in AD [2, 3]. The plaques contain the misfolding and aggregation of amyloid-β (Aβ) protein [4]. Although the exact role of Aβ protofibrils or oligomer in AD pathogenesis is not fully understood, accumulating evidence suggests that most of them are toxic species responsible for neuron dysfunction and death [5–8]. Moreover, the Aβ aggregates have already exist in the brain of an AD patient. These aggregates will continue to format neurotoxin reactive oxygen species (ROS), which will trigger a series of damages of cellular components such as DNA, lipids, and proteins and cause the oxidative stress in AD [9]. Damage to the neuron usually results in learning and memory deficits. Therefore, it is envisioned that inhibiting amyloid β aggregation and reactive oxygen species formation could be promising therapeutic targets for preventing or alleviating the pathology of AD.

Nanomaterials have unique physicochemical properties such as small size, large surface area, and high reactivity. Recently, research has emphasized that with appropriate surface modifications, nanoparticles (NPs) can be used as tools for drug delivery, imaging, and therapeutic applications in AD [10–12]. Generally, NPs have large surface area which brings great adsorption capacities. Specifically, Aβ can bind on some NPs to retard the fibrillation processes of Aβ. For example, the binding of an Aβ monomer on polyoxometalates would lower the concentration of free monomers and shift the equilibrium away from fibrillation [13]. Among these nanomaterials, selenium nanoparticles (SeNPs) display several features that make them well suited for biomedical applications, such as straight-forward preparation and stability. Selenium is an essential trace element, which plays important roles in cellular redox regulation, detoxification, and immune-system protection [14]. Therefore, comparing with inorganic and organic selenium compounds, SeNPs have better biocompatibility, and lower toxicity [15]. Nowadays, surface modifications of NPs facilitate binding affinity between NPs and Aβ and help to remedy the biochemical properties of the conjugated molecules. Our previous study found that the anti-amyloid capacity of SeNPs could be further enhanced by grafting an anti-amyloid peptide (LPFFD) onto a SeNP surface [12]. However, most of these researches did not focused on the anti-oxidation ability of NPs after surface modifications.

Chlorogenic acid (CGA) is the main polyphenol component in fruits like apple, pears, and berries and is particularly abundant in coffee [16]. CGA has a number of pharmacological properties, such as anti-cancer, anti-inflammatory, and anti-bacterial [16]. Especially, CGA has anti-oxidative and neuroprotective activities, which allows application to treat Alzheimer’s disease [17, 18]. However, the effect of CGA on Aβ aggregation has never been reported. In addition, the use of CGA is limited by its low bioavailability and stability, and only one third of CGA absorbed from the gastrointestinal tract reaches blood circulation [19]. A number of studies have reported that due to the small size of NPs, they can enter the bloodstream directly via inhalation, ingestion, and transportation throughout the circulation to many organs. Therefore, in order to resolve this issue, we have explored the binding of CGA to SeNPs (CGA@SeNPs) to improve CGA potential therapeutic efficacy. In this context, the aim of this study was to investigate the potential therapeutic efficacy of CGA@SeNPs in anti-Aβ aggregation and anti-oxidation. To the best of our knowledge, there is no previous report of using CGA@SeNPs for AD therapy.

## Methods/Experimental

### Materials and Cell Lines

Aβ40 was synthesized at GL Biochem Ltd. (Shanghai, China). Selenium dioxide (Na_2_SeO_3_), NaBH_4_, thiazolyl blue tetrazolium bromide (MTT), thioflavine T, and 2′,7′-dichlorodihydrofluorescein diacetate (DCFH-DA) were from Sigma (St. Louis, MO, USA). CGA was purchased from Aladdin (Shanghai, China). Dulbecco’s modified Eagle medium (DMEM), fetal bovine serum (FBS), and horse serum were obtained from Gibco (Life Technologies AG, Switzerland). PC12 cells (rat pheochromocytoma, American Type Culture Collection) were cultured in DMEM medium supplemented with 5% FBS and 10% horse serum at 37 °C in a 5% CO_2_ humidified environment at 37 °C.

### Preparation of CGA@SeNPs

First, stock solutions of 25 mM CGA, 0.1 M Na_2_SeO_3_, and 0.1 M NaBH_4_ were prepared. Then, 200 μL aliquot of Na_2_SeO_3_ solution was mixed with varied volumes of CGA, and the reactant concentration ratios of Na_2_SeO_3_ to CGA were 1:1, 1:2, 1:4, 1:6, and 1:8. After that, 200 μL of 0.1 M NaBH_4_ was added into the mixture and stirred for 30 min. The color of the solution was changed to red. Excess CGA and Na_2_SeO_3_ were removed by dialysis. We found that the best concentration ratio of Na_2_SeO_3_ to CGA was 1:6.

### Characterization of CGA@SeNPs

The as-prepared CGA@SeNPs were characterized by transmission electron microscope (TEM; Hitachi, H-7650), Fourier transform infrared spectroscopy (FT-IR; Equinox 55 IR spectrometer), and UV-vis spectroscopy (Carry 5000 spectrophotometer). The size distribution was determined by Zetasizer Nano ZS particle analyzer (Malvern Instruments Limited). The fluorescence of nanoparticles was carried out with a JASCO FP6500 spectrofluorometer (*λ*ex = 350 nm).

### H_2_O_2_ Generation Assay

The hydroxyl radical, 2, 29-azinobis-(3-ethylbenzothiazoline-6-sulfonic acid) (ABTS^+^), and superoxide anion scavenging activities of CGA and CGA@SeNPs were analyzed by commercial kits (Nanjing Jiancheng Bioengineering Institute, Nanjing, China). The reducing power of the samples was measured as follows: 2 mL of CGA or CGA@SeNPs was incubated with 2 mL of phosphate buffer (0.2 mol/L, pH 6.6) and 2 mL of K_3_Fe(CN)_6_ (1%, *w*/*v*) at 50 °C for 20 min. After that, the reaction was stopped by adding 2.5 mL of trichloroacetic acid (10%, *w*/*v*) and centrifuged at 3000 rpm for 10 min. Two milliliters of the supernatant was mixed with 2 mL of distilled water and 1 mL of FeCl_3_ (0.1%, *w*/*v*) at room temperature for 10 min. Finally, the absorbance was measured at 700 nm. Vitamin c (Vc) was used as a positive control in anti-oxidation activity assay.

The H_2_O_2_ generation in Aβ40 was analyzed by DCFH-DA. DCFH-DA stock solution (1 mM) purchased from Beyotime Institute of Biotechnology (Shanghai, China). Four micromolars of horseradish peroxidase (HRP) was prepared in buffer (20 mM Tris-HCl/150 mM NaCl, pH 7.4). Sample solutions containing 35 μM Aβ40 with or without 20, 40, and 60 μg/mL CGA@SeNPs/CGA were incubated at 37 °C for 3 days. Ascorbate (10 μM) was added to each sample and further incubated for 1 h. Sample analyses were performed by adding DCFH-DA (20 μL, 100 μM) and HRP (2 μL, 0.04 μM) to the sample solution (10 μL). Fluorescence spectra with excitation and emission wavelengths of 488 and 525 nm were measured by a JASCO FP6500 spectrofluorometer.

### Thioflavine T Fluorescence Measurements

The kinetics of Aβ40 fibrillation was detected by using the dye thioflavine T (ThT). Briefly, 35 μM Aβ40 was incubated with 20, 40, and 60 μg/mL of CGA@SeNPs/CGA at 37 °C from 0 to 5 days. Each day, 50 μL of solution was taken out and added into 200 μL ThT solution (15 μM ThT in 50 mM PBS, pH 7.4). Then, the excitation of ThT was 440 nm, and the emission wavelength of 490 nm was recorded.

### TEM

Morphologies of Aβ40 in the presence or absence of CGA@SeNPs/CGA were observed by TEM (Hitachi, H-7650). The samples were prepared in the same way as in the ThT fluorescence assay. After 3 days of incubation, 10 μL of sample solution was spotted on the carbon-coated copper grids for 10 min. Then, each grid was stained with 1.5% (*w*/*v*) phosphotungstic acid (pH 7.4) and allowed to dry.

Binding of nanoparticles to Aβ40 was also confirmed by TEM. Firstly, 35 μM Aβ40 was incubated for 3 days to format the fibers. Then, 20 μg/mL nanoparticles was added into the pre-incubated solution and incubated for another 6 h. After that, this sample was examined on TEM.

### Resonance Light Scattering Measurements

The resonance light scattering (RSL) was measured according to the method of Yu et al. [20] with some modifications. A certain amount of Aβ40 dispersion was diluted to 1 mL with H_2_O, while the final concentration of CGA@SeNPs varied from 0.05 to 0.45 μg/mL. The mixture was incubated for 10 min. The RLS intensities were recorded on Cary Eclipse fluorospectrophotometer (Agilent technologies, USA) by synchronously scanning the excitation and emission monochromators (Δ*λ* = 0 nm) between 200 and 800 nm. Both the excitation and emission slit width were set to 5 nm.

### Cytotoxicity Assay

The cell viability was analyzed by using MTT assay. PC12 cells were plated at a density of 5000 cells per well on 96-well plates in fresh medium without FBS. Aβ (35 μM) was co-incubated with or without 60 μg/mL CGA@SeNPs/CGA for 3 days. After that, the cells were treated with these samples for another 72 h. After incubation, the cells were treated with 10 μL MTT per well, following the manual of Cell Titer 96 Aqueous One Solution Cell Proliferation assay (Promega). Release of lactate dehydrogenase (LDH) was assayed using the commercial detection kit. The PC12 cells were treated as above. At the end of the treatments, the 96-well plate was centrifuged at 1500×*g* for 10 min. The LDH activity in supernatants was measured according to the manufacturer’s instructions.

### Intracellular ROS Generation

The effects of CGA@SeNPs/CGA on Aβ40-induced intracellular ROS generation were monitored by DCFH-DA assay. The PC12 cells (1 × 10^5^ per well) were treated in the same way as in the MTT assay. Then, the cells were washed twice with PBS and incubated with DCFH-DA (10 mM) at 37 °C for 30 min. The intracellular ROS level was examined under fluorescent microscope (magnification ×200) with the excitation and emission wavelengths at 488 nm and 525 nm, respectively. To measure the intracellular ROS level, the cells were treated in the same way and harvested by centrifugation, re-suspended in PBS. Then, the cells were analyzed by flow cytometry.

### TUNEL-DAPI Co-staining Assay

The PC12 cells were treated in the same way as in the MTT assay. After that, cells in chamber slides were fixed with 3.7% form-aldehyde and permeabilized with 0.1% Triton X-100 in PBS. The staining assay was carried out by using terminal transferase dUTP nick end labeling (TUNEL) assay kit (KeyGen BioTECH, Nanjing, China) following the manufacturer’s protocol. Images were captured using fluorescent microscope (magnification ×200).

### Statistics

Statistical significance was estimated using one-way analysis of variance (ANOVA) followed by Bonferroni’s post hoc test. Statistical significance was set at *P* < 0.05.

## Results and Discussion

### Characterization of CGA@SeNPs

**Fig. 1:**
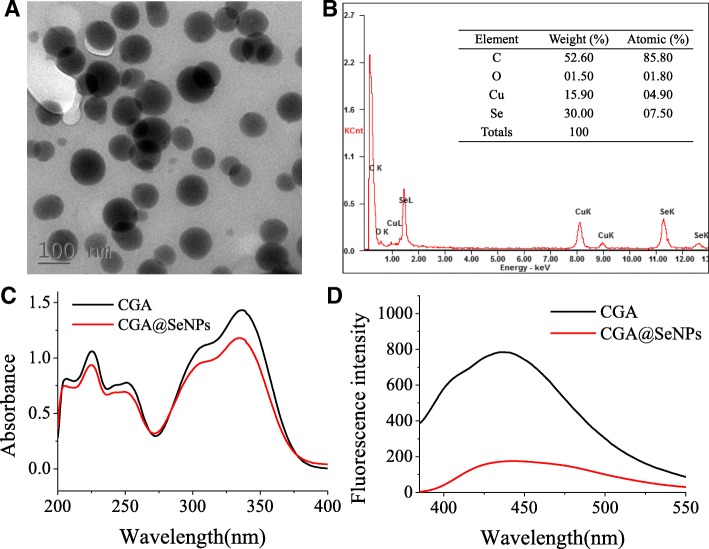
Characterization of CGA@SeNPs. **a** TEM images of CGA@SeNPs. **b** EDX analysis of CGA@SeNPs. **c** UV-vis absorption spectrum of CGA@SeNPs. **d** The emission spectrum of CGA@SeNPs

In this study, we use a simple method to synthesize CGA@SeNPs by reducing a mixture of CGA and Na_2_SeO_3_ with NaBH_4_. Nanoparticles with size ranging from 30 to 150 nm were more favorable for cellular uptake [21]. TEM showed CGA@SeNPs have a spherical structure with a diameter about 100 nm (Fig. 1a), suggesting that the size of CGA@SeNPs was suited for the biological application. Elemental composition analysis showed that Se, C, and O can be easily found in the energy-dispersive X-ray spectroscopy (EDX) graph of CGA@SeNPs (Fig. 1b). The signal of the Se atoms was 30.00%, together with the strong C atom signal (52.60%) and O atom signal (1.50%) from CGA, indicating that we have successfully prepared the CGA@SeNPs.

The UV-vis absorption spectrum of CGA shows a characteristic absorption peak at 334 nm (Fig. 1c). However, a slight shift was observed in the UV-vis absorption spectrum of CGA@SeNPs and the absorption peak was changed to 337 nm. The emission wavelengths of CGA and CGA@SeNPs were detected by fluorescence spectrophotometer. The emission wavelength of CGA was 442.9 nm, which was shifted to 437.5 nm in CGA@SeNPs (Fig. 1d).These results confirmed the presence of CGA on the surface of SeNPs. The FT-IR spectrum of CGA@SeNPs gives evidence that CGA has formed a part of the nanocomposite. The O–H stretching frequency is located at 3353.99 cm^−1^ in the FT-IR spectrum of CGA (Additional file 1: Figure S1); however, this characteristic peak in CGA@SeNPs was changed to 3419.00 cm^−1^. The shift confirmed that CGA was conjugated to the surface of SeNPs through the −OH group.

The stability of CGA@SeNPs under physiological conditions is important for evaluating their future applications. Thus, the size distribution of CGA@SeNPs in PBS (pH 7.4) was monitored at room temperature for 7 days. As shown in Additional file 1: Figure S2, the size of CGA@SeNPs remained stable with an average size about 100 nm, and CGA@SeNPs retained good water dispersibility in PBS within 7 days. The favorable stability of CGA@SeNPs supports their potential application in the medical area.

### Inhibition of ROS Generation

The deposited Aβ can activate microglia and stimulate microglia to produce neurotoxins, such as ROS, which can further cause severe neuronal damage in the brain [22]. Besides, ROS such as hydrogen peroxide (H_2_O_2_) can also be generated by Aβ aggregates [23]. Thus, we investigated the antioxidant activities of CGA@SeNPs and the effect of CGA@SeNPs on Aβ aggregate-induced H_2_O_2_ formation. As shown in Additional file 1: Figure S3, the CGA@SeNPs have strong scavenging activities against radicals in a concentration-dependent manner. When the concentration reached to 60 μg/mL, the hydroxyl radical, ABTS^+^, and superoxide anion scavenging activities reached to 70.3%, 95.2%, and 95.1%, respectively. Obviously, the reducing power of CGA@SeNPs increased with increasing concentrations. Among all samples, CGA@SeNPs exhibited strong reducing power and scavenging capacities on hydroxyl radical, ABTS^+^, and superoxide anion than Vc and CGA. These results suggested that CGA@SeNPs showed higher antioxidant activity, which may help in scavenging active oxygen in AD. The point to be noted is that the modification of CGA on SeNPs exerts a synergistic effect on antioxidant activity.

**Fig. 2:**
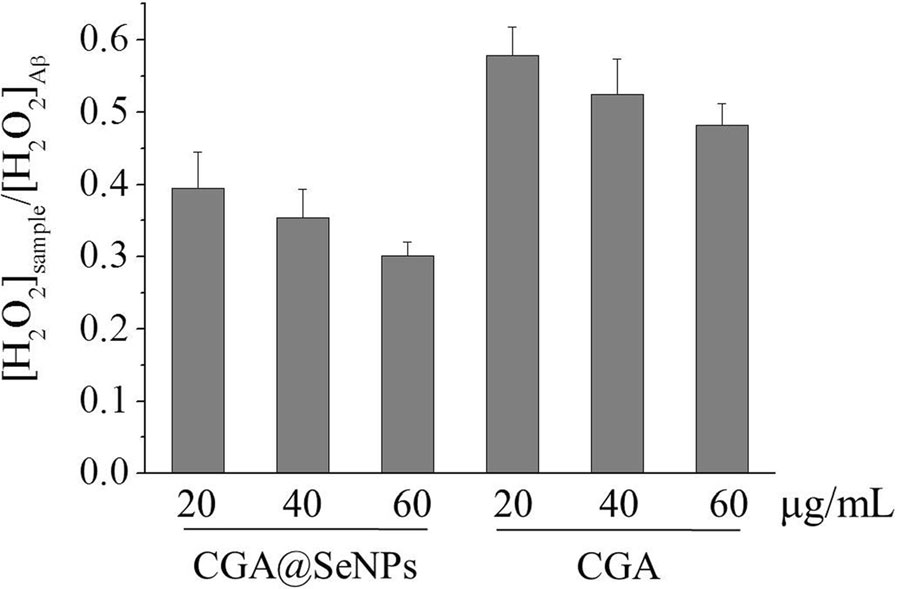
H_2_O_2_ generation from reactions of Aβ40 in the presence or absence of nanoparticles. Aβ = 35 μM, nanoparticles/CGA = 20, 40, and 60 μg/mL

The effect of CGA@SeNPs on the Aβ-mediated H_2_O_2_ generation was investigated by a DCFH-DA assay [24], which can indicate the generation of H_2_O_2_ from the Aβ in the presence or absence of CGA@SeNPs. As shown in Fig. 2, CGA@SeNPs and CGA decreased H_2_O_2_ in a dose-dependent manner. More importantly, the Aβ samples containing CGA@SeNPs show less H_2_O_2_ than those containing CGA, revealing that high antioxidant activity of CGA@SeNPs showed more effect than CGA in reducing the Aβ-induced H_2_O_2_ generation.

### Inhibition of Aβ Aggregation by CGA@SeNPs

**Fig. 3:**
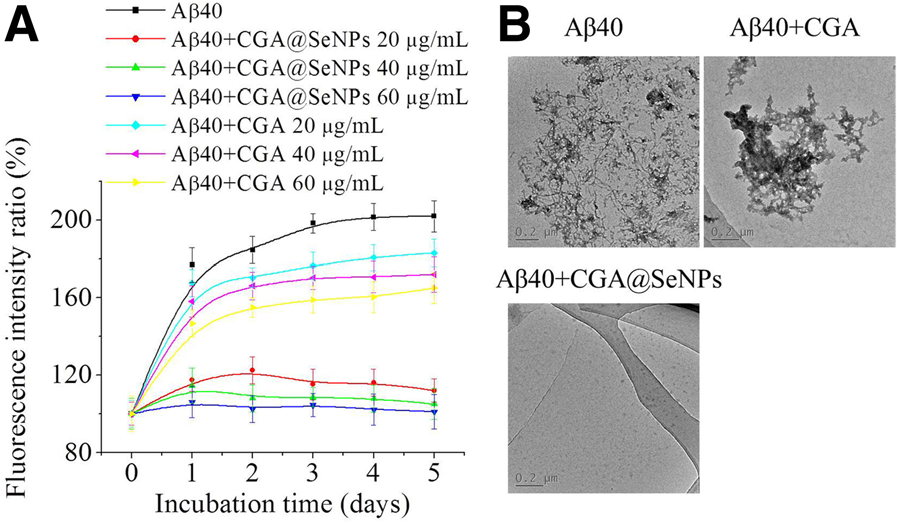
Inhibition effect of CGA@SeNPs on Aβ40 fibrillation. **a** ThT fluorescence of Aβ40 fibril formation in the presence or absence of nanoparticles/CGA from 0 to 5 days. Aβ40 = 35 μM, nanoparticles/CGA = 20, 40, and 60 μg/mL. **b** Morphology of Aβ40 incubated with or without nanoparticles or CGA for 3 days. Aβ40 = 35 μM, nanoparticles/CGA = 60 μg/mL

Although the antioxidant activity of CGA is a well-known property, the anti-amyloid aggregation activity of CGA is still unknown. To verify the feasibility of the CGA@SeNPs for AD therapeutic applications, the inhibition effect of CGA@SeNPs on Aβ aggregation was first investigated by ThT-based fluorometric assay, which is a well-established method to monitor the β-sheet formation under continuous time [25]. As shown in Fig. 3a, Aβ40 aggregated spontaneously and the fluorescence of Aβ fibrils increased gradually until it reached a plateau by 3 days of incubation. We have observed that CGA@SeNPs were able to slowly aggregate amyloid proteins with the increasing of concentration. Fluorescence intensity of Aβ40 fibers about two times more than that of Aβ40 incubated with 60 μg/mL CGA@SeNPs was achieved. However, little or no change in ThT fluorescence intensity was observed in CGA, suggesting CGA only slightly inhibited the Aβ40 aggregation.

The morphological changes of Aβ40 incubated with or without CGA or CGA@SeNPs are shown in Fig. 3b. Aβ40 incubated in 3 days formed abundant fibrils, whereas in the presence of CGA@SeNPs (60 μg/mL), no fibrils formed. As expected, CGA did not significantly inhibit Aβ fibrillogenesis, whereas large amounts of aggregates were observed in the solution of CGA-treated Aβ40, which was consistent with ThT fluorometric assay. These results indicated that after modification of CGA on SeNPs, it could obviously decrease β-sheet formation.

### Binding Activity of CGA@SeNPs to Aβ40

**Fig. 4:**
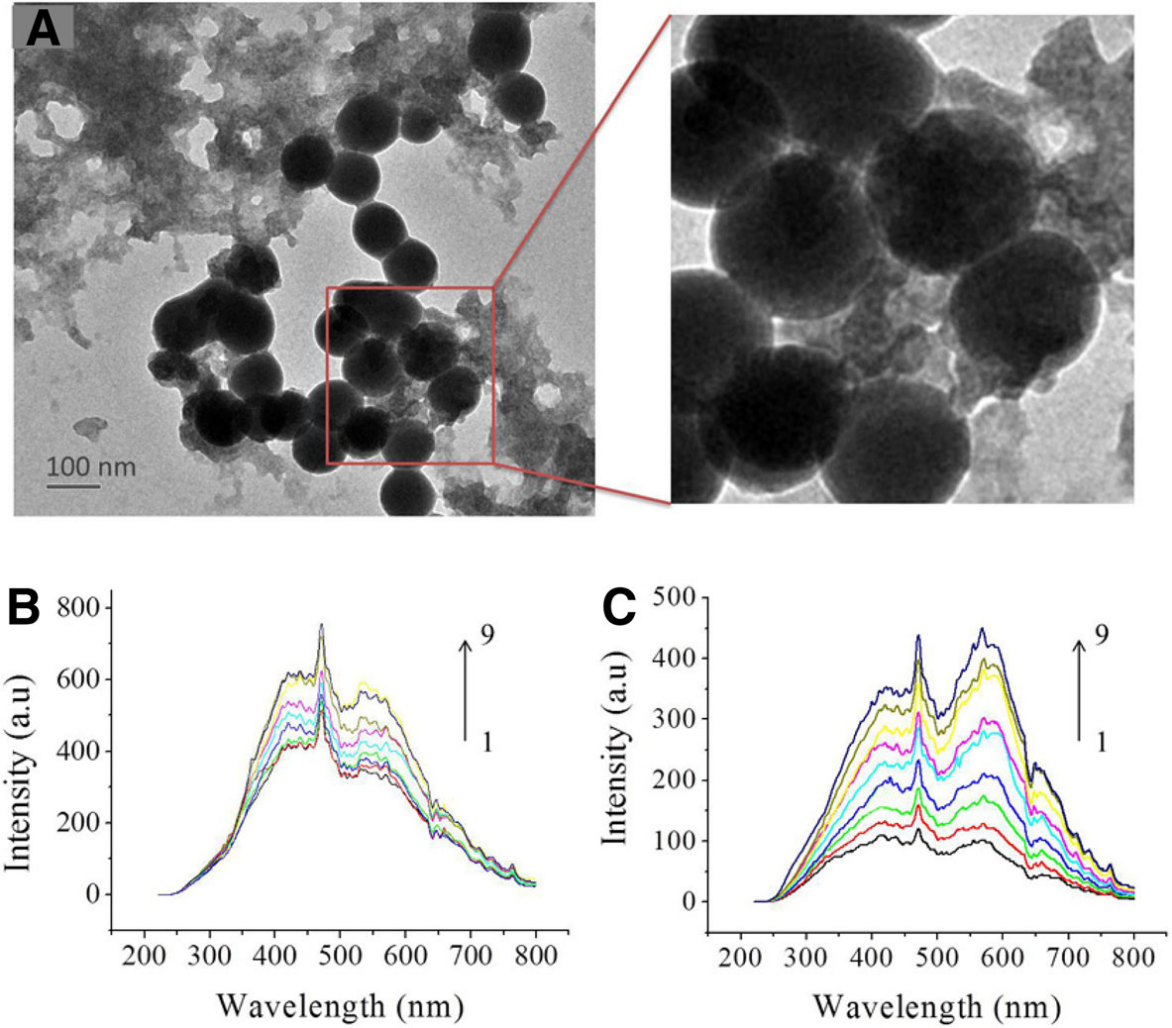
Affinity of CGA@SeNPs for Aβ40. **a** The binding capacity of CGA@SeNPs on Aβ40 fibers. The performed fibers of Aβ40 were incubated with CGA@SeNPs and examined on TEM. Aβ40 = 35 μM, CGA@SeNPs = 60 μg/mL. **b** Affinity and specify of CGA@SeNPs for Aβ40 monomer. The RLS spectra of Aβ40 monomer in the presence of different concentration of CGA@SeNPs. Aβ40 = 600 nM, nanoparticles concentration, 1–9: 0, 0.05, 0.1, 0.15, 0.2, 0.25, 0.3, 0.35, and 0.4 μg/mL. **c** The RLS spectra of CGA@SeNPs, nanoparticles concentration, 1–9: 0.05, 0.1, 0.15, 0.2, 0.25, 0.3, 0.35, 0.4, and 0.45 μg/mL

To gain a better understanding of the inhibitory activity of CGA@SeNPs on Aβ aggregation, we investigated the binding affinity of CGA@SeNPs for Aβ40. First, to evaluate the binding of nanoparticles on Aβ40 fibers at the ultrastructural level, Aβ40 fibers were incubated with CGA@SeNPs. We observed that the fibrils specifically bound on the surface of SeNPs without the presence of any free unassociated suspended nanoparticles (Fig. 4a).

Resonance light scattering (RLS) is a powerful optical technique based on elastic light scattering and has been widely applied to study the size, shape, and distribution of nanoparticles in solution [26]. Thus, we next used RLS to analyze the interaction between Aβ40 monomer and CGA@SeNPs. As shown in Fig. 4b, c, the RLS intensity of NPs is much lower than that of NPs incubated with 600 nM Aβ40 monomer at the same concentration. Once the CGA@SeNPs were exposed to Aβ molecules, Aβ is believed to bind on the surface of SeNPs via N-donors containing side chains of amino acids to form a Se–N bond [27]. The formation of conjugates of Aβ molecules on CGA@SeNPs results in increasing the size of scatters, which enhanced the RLS intensity of the system (Fig. 4b). Combined with the result of ThT assay and TEM, it is speculated that the interactions between the large CGA@SeNP surface and Aβ40 monomer could result in unfavorable conditions for nucleation and fibril growth through the blocking of direct contact between monomers. Thus, CGA@SeNPs exert more effect than CGA in inhibiting the Aβ40 aggregation.

### Inhibition of Aβ40 Neurotoxicity

**Fig. 5:**
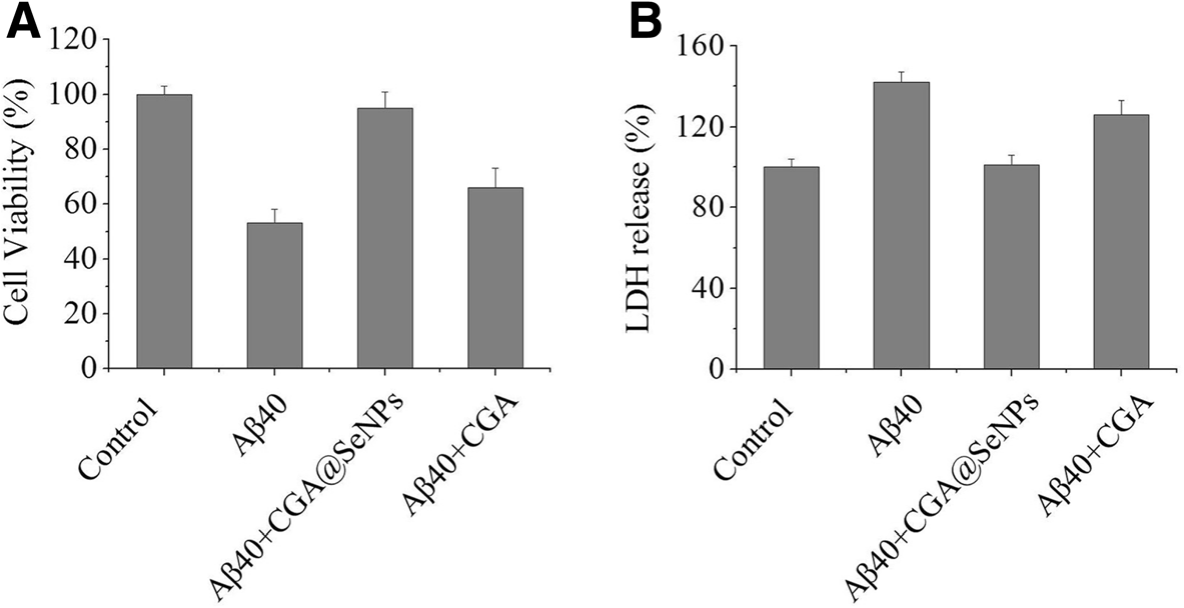
The neurotoxicity of Aβ40 incubated with or without CGA@SeNPs/CGA. **a** The cell viability of PC12 cells toward Aβ40 in the presence of CGA@SeNPs or CGA tested by MTT assay. **b** The release of LDH into the culture medium. Aβ40 = 35 μM, CGA@SeNPs/CGA = 60 μg/mL

The cytotoxicity of Aβ40 in the presence or absence of CGA@SeNPs in PC12 cells was investigated by MTT assay. Figure 5a shows that Aβ40 fibers were toxic to the PC12 cells, which reduced cell viability to 53%. In the presence of CGA@SeNPs, the survival of the cells increased to about 95%. However, pretreatment of the cells with CGA only slightly increased the survival of the cells to 66%. Furthermore, the CGA@SeNPs were non-toxic to PC12 cells (Additional file 1: Figure S4), suggesting that CGA@SeNPs could inhibit neurotoxicity of Aβ40.

It has been reported that Aβ preferentially immobilized on the cell surface and caused the cell membrane damage [28]. Therefore, to determine the cell membrane integrity after exposure to Aβ40 in the presence or absence of NPs/CGA, we measured the extracellular LDH levels in cell culture medium. In this assay, the high level of LDH reflects the damage of cell membrane. As shown in Fig. 5b, compared to the control, after exposure to Aβ40, LDH release increased to 142%. In accordance with the result of MTT, in the presence of CGA@SeNPs, LDH release was reduced to a normal level. Interestingly, we observed that CGA did not reduce the LDH concentration, suggesting that CGA cannot prevent PC12 cells from cell membrane damage induced by Aβ aggregates.

### Prevention of Aβ40-Induced ROS Generation in PC12 Cells

Aβ aggregates are capable of producing ROS which could cause oxidative stress in AD [29]. Due to the anti-oxidation and anti-aggregation properties of CGA@SeNPs, we hypothesized that it would be able to reduce Aβ40-induced ROS generation in PC12 cell. Thus, the effect of NPs on the Aβ40 aggregate-induced ROS generation was measured by DCFH-DA. DCF is a fluorescent marker derived from the reaction of nonfluorescent DCFH-DA with ROS, which can indicate the generation of ROS induced by Aβ40 aggregates. As shown in Additional file 1: Figure S5, treatment with Aβ40 aggregates caused more than a 1.5 fold increase in ROS content relative to untreated cells. CGA@SeNP treatment decreased the DCF fluorescence intensity in PC12 cells, indicating that the CGA@SeNPs decreased the Aβ fibril-induced ROS to a normal level. Interestingly, CGA also reduced the ROS generation in PC12 cells, but not as obviously as CGA@SeNPs.

### Prevention of Aβ40-Induced Cell Apoptosis in PC12 Cells

**Fig. 6:**
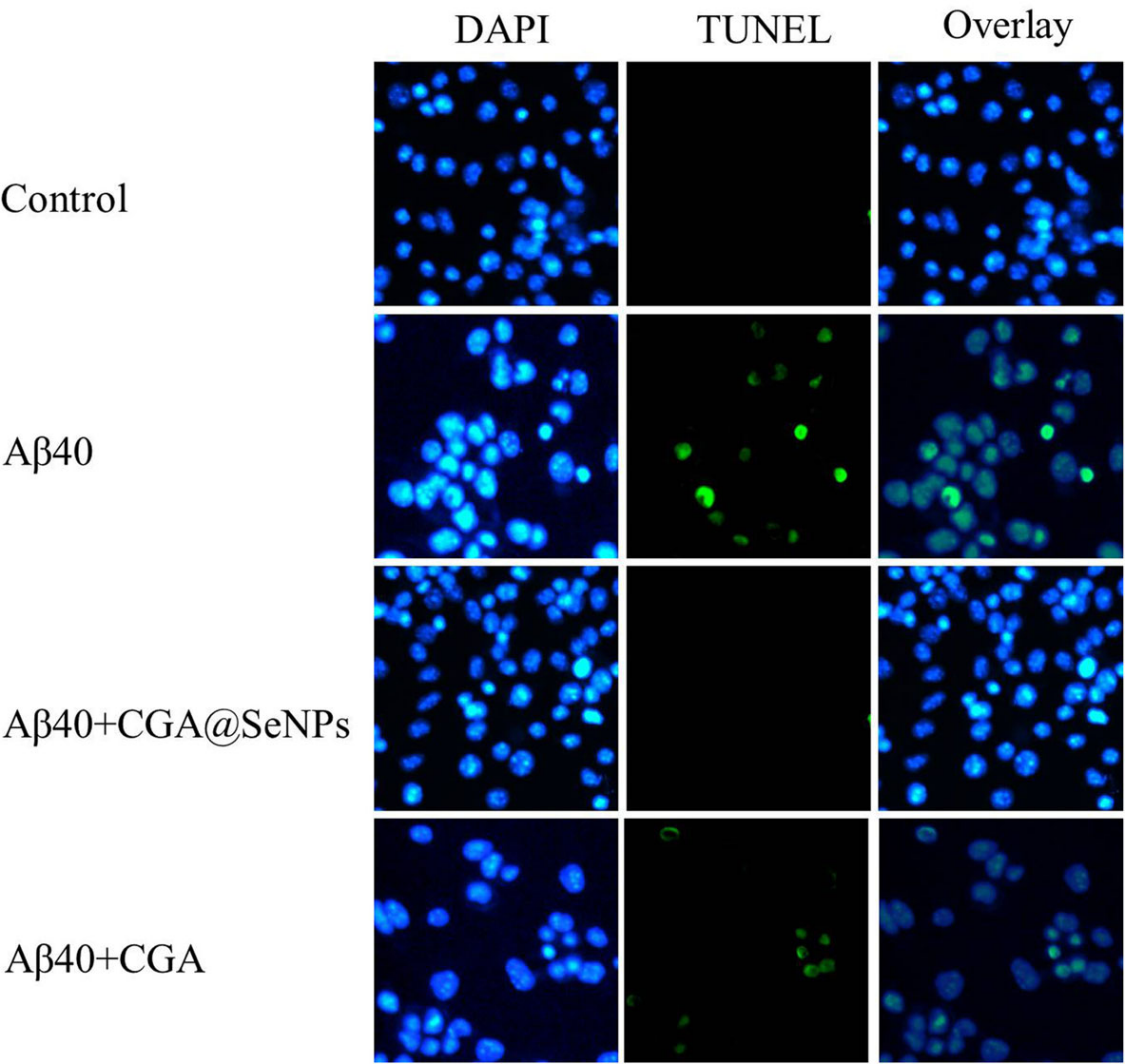
CGA@SeNPs prevent Aβ40-induced cell apoptosis in PC12 cells. Cell apoptosis was detected by TUNEL-DAPI co-staining assay. Normal cell nucleus was stained in blue, and DNA fragment was stained in green. Aβ40 = 35 μM, CGA@SeNPs/CGA = 60 μg/mL

It has been reported that the death of neuronal cells caused by Aβ fibril is a critical event in AD pathology [30]. To further study the cytotoxicity of Aβ40 fibrils, we performed TUNEL-DAPI co-staining to determine the effect of NPs or CGA in Aβ40-induced cell apoptosis. DNA fragmentation is a hallmark of cell apoptosis, which could be detected at the early stages of apoptosis by using TUNEL [31]. As shown in Fig. 6, the apoptotic nuclei were stained in bright fluorescent green by TUNEL. Aβ40 dramatically increased the number of TUNEL-stained nuclei, whereas CGA@SeNP treatment caused a decrease of TUNEL-positive nuclei, demonstrating that CGA@SeNPs could protect neurons from Aβ40-induced PC12 cell apoptosis. Otherwise, high levels of ROS in cells also participate in apoptosis [32]. Thus, these results connoted that Aβ40-aroused apoptosis might be attributed to the generation of ROS. It is worth noticing that CGA can also reduce the Aβ40-induced cell apoptosis. From MTT and LDH assay, CGA did not enhance the survival of the cell and decrease the Aβ aggregate-induced cell membrane damage. Combined results of TEM and ThT assay, we found that CGA has anti-oxidation property which can clear active oxygen in the process of Aβ aggregation, but it can not entirely inhibit the Aβ aggregation. Therefore, CGA could decrease ROS generation in incubation solution and PC12 cells, protecting ROS-induced cell apoptosis, but it cannot decrease the Aβ aggregate-induced cell membrane damage. The cell membrane damage will result in the leakage of intracellular contents into the surrounding tissue, which could lead to tissue damage and inflammation [33]. Thus, combination of inhibited Aβ aggregation and reactive oxygen species formation could be considered as a new therapeutic method.

## Conclusions

In summary, CGA has an anti-oxidation pharmacological property, but it has no effect in inhibiting Aβ fibrillation. Surface modification of CGA on SeNPs gives it a new anti-aggregation property. Aβ40 could bind on the surface of CGA@SeNPs, which results in unfavorable conditions for nucleation and fibril growth through the blocking of direct contact between monomers. In that case, CGA@SeNPs inhibited Aβ40 aggregation, cleared the ROS, and protected PC12 cells from the cell membrane disruption and ROS-mediated apoptosis induced by Aβ40 aggregates. However, in CGA group, the Aβ aggregates immobilized on the cell surface and caused the cell membrane damage, which also result in cell death. Overall, CGA@SeNPs are more efficient than CGA in reducing Aβ40 toxic in long-term use.

## Additional file

Additional file 1: **Figure S1.** FT-IR spectra of CGA and CGA@SeNPs. Nanoparticles were centrifuged at 10000*g* for 15 min, and the sediment was dried in room temperature. **Figure S2.** The stability of CGA@SeNPs in PBS (pH = 7.4) for 7 days (A). CGA@SeNPs dispersed in PBS after 7 days of being stored. **Figure S3.** Anti-oxidation property of CGA@SeNPs. A. OH radical scavenging activity of CGA@SeNPs. B. ABTA^+^ scavenging activity of CGA@SeNPs. C. Superoxide anion scavenging activity of CGA@SeNPs. D. The reducing power of CGA@SeNPs. Vc was used as positive control. **Figure S4.** The neurotoxicity of CGA@SeNPs and CGA. **Figure S5.** CGA@SeNPs reduced intracellular ROS formation in PC12 cells (A). Quantitative analysis of DCF fluorescence intensity of PC12 cells treated with Aβ40 alone or in the presence of CGA@SeNPs/CGA by a flow cytometer (B). Aβ40 = 35 μM, CGA@SeNPs/CGA = 60 μg/mL. (DOC 1592 kb)

## Abbreviations

ABTS^+^: 2, 29-Azinobis-(3-ethylbenzothiazoline-6-sulfonic acid)
AD: Alzheimer’s disease
Aβ: Amyloid-β
CGA: Chlorogenic acid
DCFH-DA: 2′,7′-Dichlorodihydrofluorescein diacetate
DMEM: Dulbecco’s modified Eagle medium
EDX: Energy-dispersive X-ray spectroscopy
FBS: Fetal bovine serum
FT-IR: Fourier transform infrared spectroscopy
HRP: Horseradish peroxidase
LDH: Lactate dehydrogenase
MTT: Thiazolyl blue tetrazolium bromide
ROS: Reactive oxygen species
RSL: Resonance light scattering measurements
SeNPs: Selenium nanoparticles
TEM: Transmission electron microscope
ThT: Thioflavine T
TUNEL: Terminal transferase dUTP nick end labeling
Vc: Vitamin c
