# Supplementary material for: Enhanced Effect of Combining Chlorogenic Acid on Selenium Nanoparticles in Inhibiting Amyloid β Aggregation and Reactive Oxygen Species Formation In Vitro
Source: Nanoscale Res Lett. 2018 Sep 29;13:303. doi: 10.1186/s11671-018-2720-1 (PMC6163123; doi:10.1186/s11671-018-2720-1)
Supplement: Supplementary file 1 — Figure S1. FT-IR spectra of CGA and CGA@SeNPs. Nanoparticles were centrifuged at 10000g for 15 min, and the sediment was dried in room temperature. Figure S2. The stability of CGA@SeNPs in PBS (pH = 7.4) for 7 days (A). CGA@SeNPs dispersed in PBS after 7 days of being stored. Figure S3. Anti-oxidation property of CGA@SeNPs. A. OH radical scavenging activity of CGA@SeNPs. B. ABTA+ scavenging activity of CGA@SeNPs. C. Superoxide anion scavenging activity of CGA@SeNPs. D. The reducing power of CGA@SeNPs. Vc was used as positive control. Figure S4. The neurotoxicity of CGA@SeNPs and CGA. Figure S5. CGA@SeNPs reduced intracellular ROS formation in PC12 cells (A). Quantitative analysis of DCF fluorescence intensity of PC12 cells treated with Aβ40 alone or in the presence of CGA@SeNPs/CGA by a flow cytometer (B). Aβ40 = 35 μM, CGA@SeNPs/CGA = 60 μg/mL. (DOC 1592 kb) [file 11671_2018_2720_MOESM1_ESM.doc]

**Additional file**

**Enhanced effect of combining chlorogenic acid on selenium nanoparticles in inhibiting amyloid β aggregation and reactive oxygen species formation in vitro**

**Licong Yang, Na Wang, Guodong Zheng***

Jiangxi Key Laboratory of Natural Product and Functional Food, College of Food Science and Engineering, Jiangxi Agricultural University, Nanchang, 330045, China

Corresponding to: Dr. Guodong ZHENG, Jiangxi Key Laboratory of Natural Product and Functional Food, College of Food Science and Engineering, Jiangxi Agricultural University, Nanchang, 330045, China. Tel & Fax: 86-791-83813863, E-mail: zrs150716@aliyun.com


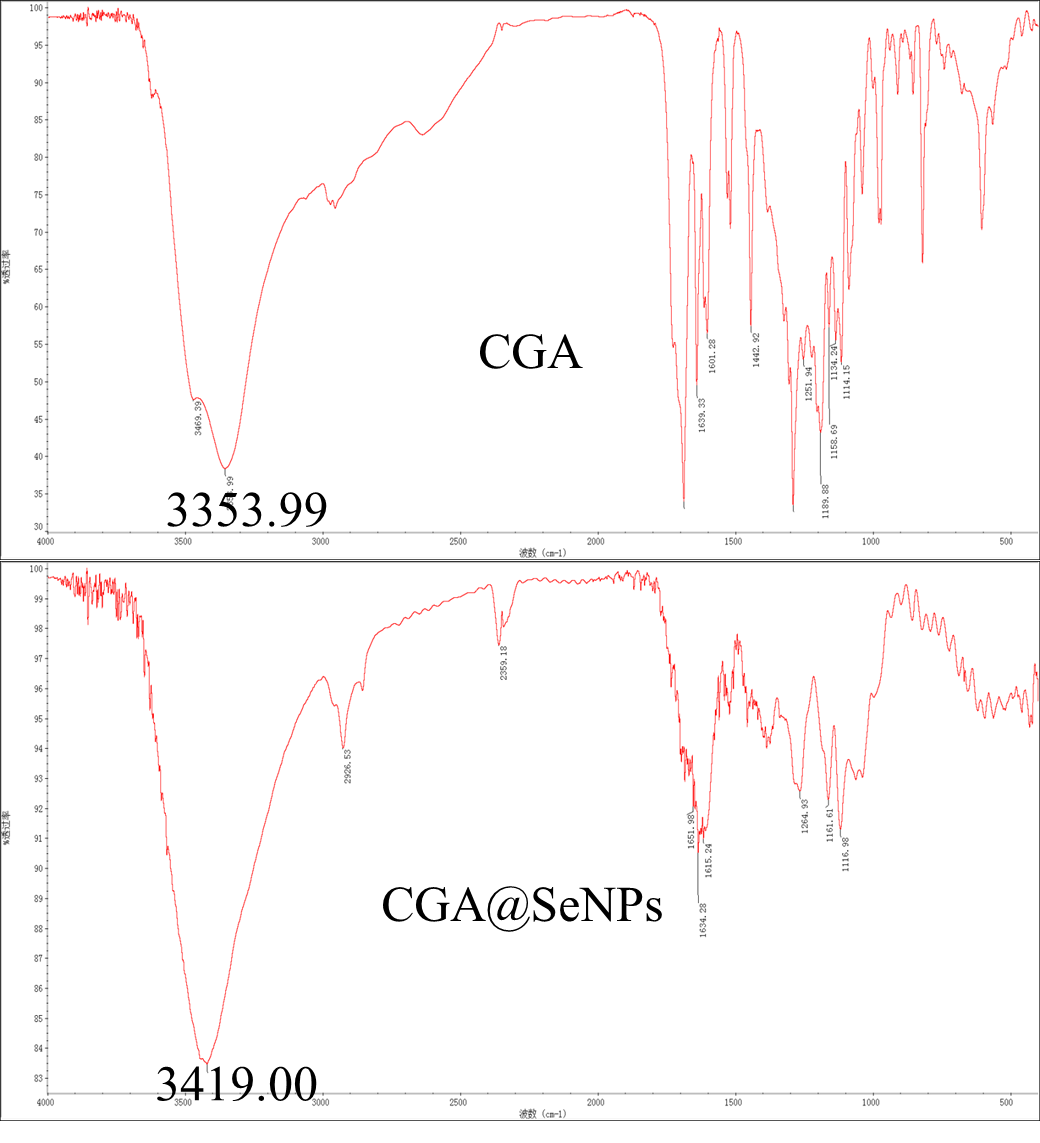


Figure S1. FT-IR spectra of CGA and CGA@SeNPs. Nanoparticles were centrifuged at 10000 g for 15 min and the sediment was dried in room temperature.


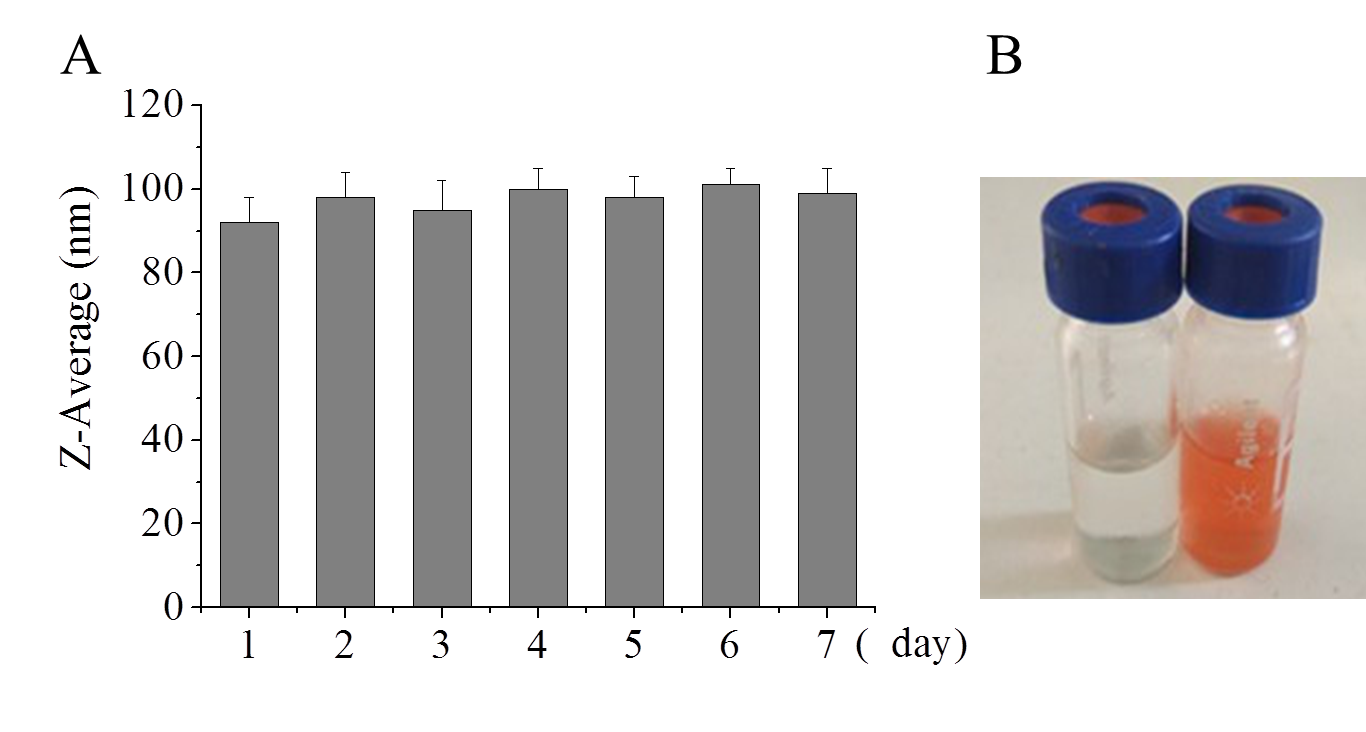


Figure S2. The stability of CGA@SeNPs in PBS (pH = 7.4) for 7 days (A). CGA@SeNPs dispersed in PBS after 7 days store.


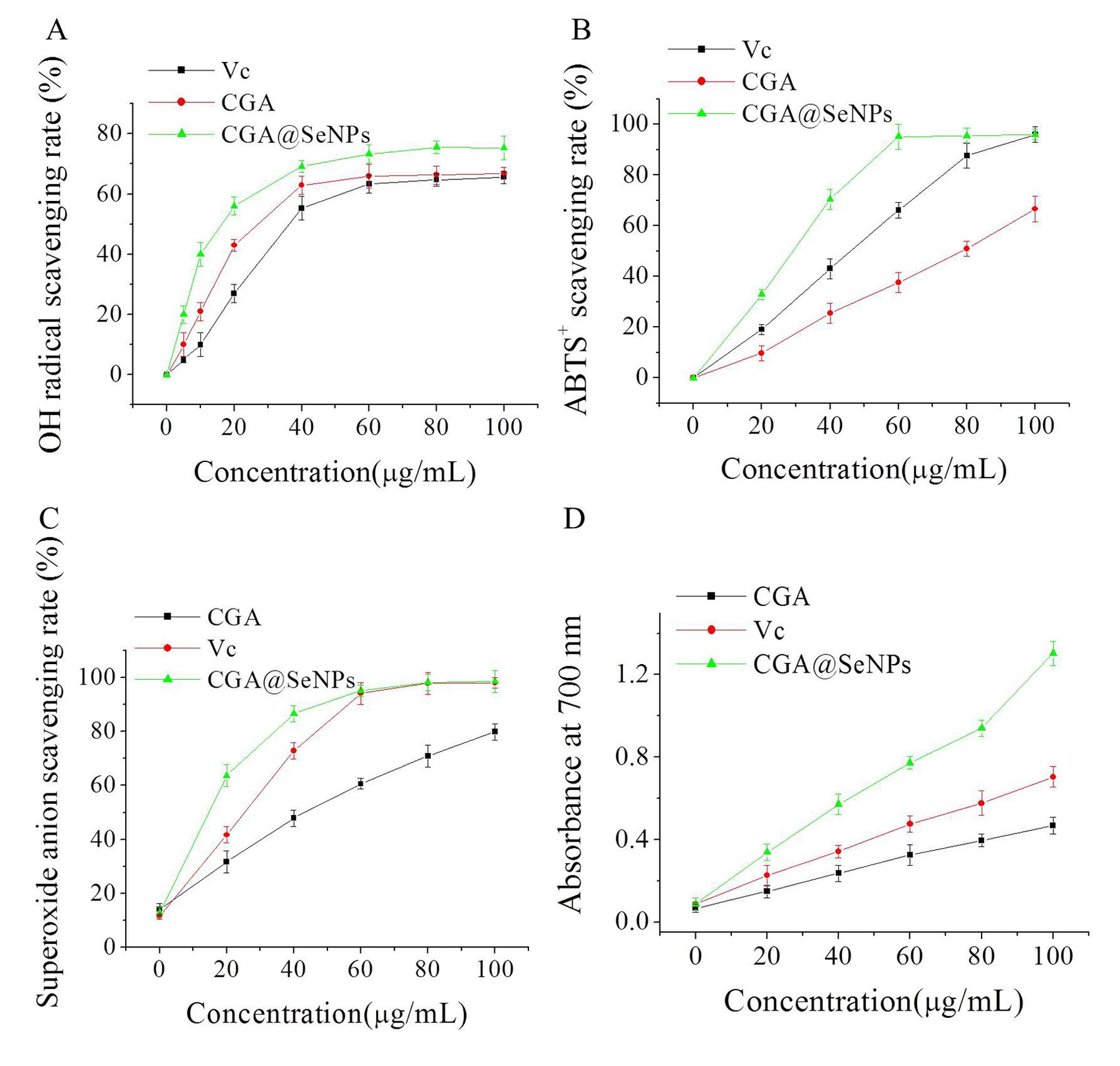


Figure S3. Anti-oxidation property of CGA@SeNPs. A. OH radical scavenging activity of CGA@SeNPs. B. ABTA^+^ scavenging activity of CGA@SeNPs. C. Superoxide anion scavenging activity of CGA@SeNPs. D. The reducing power of CGA@SeNPs. Vc was used as positive control.


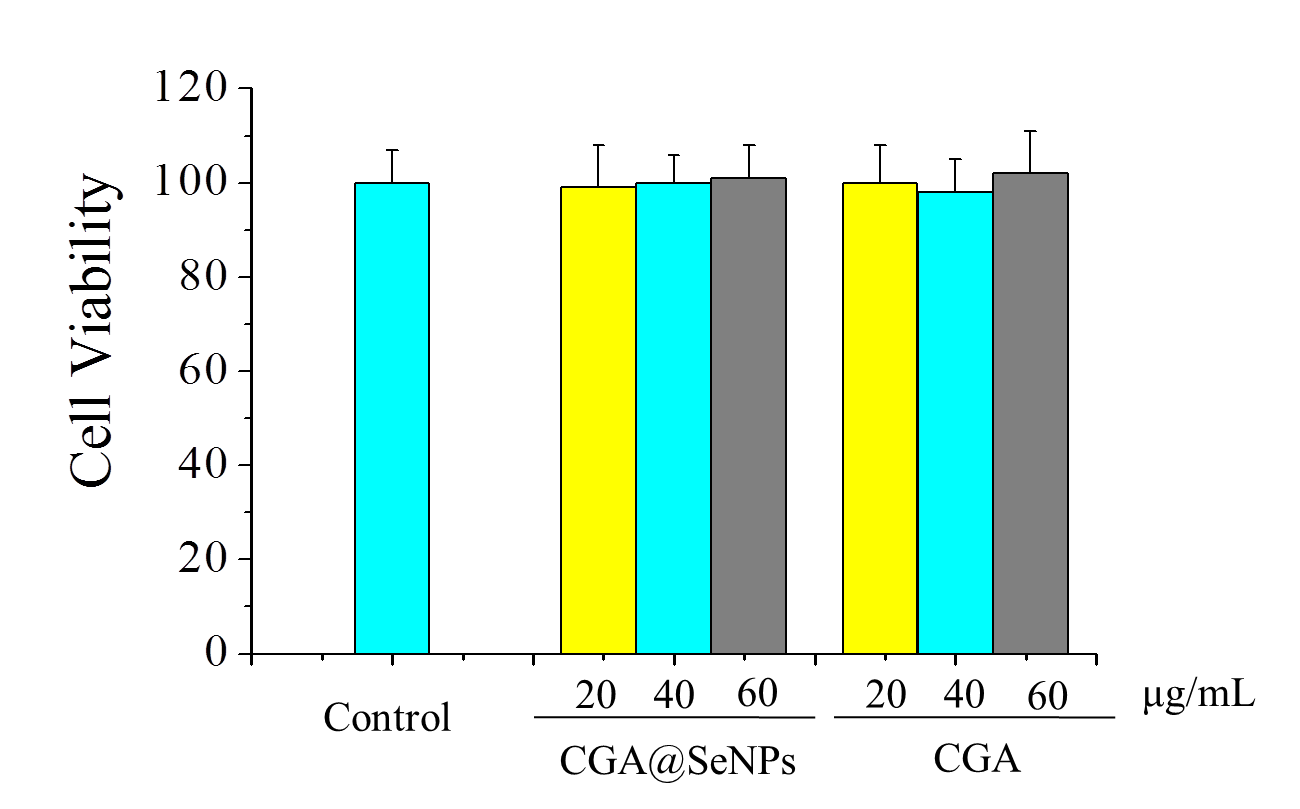


Figure S4. The neurotoxicity of CGA@SeNPs and CGA.


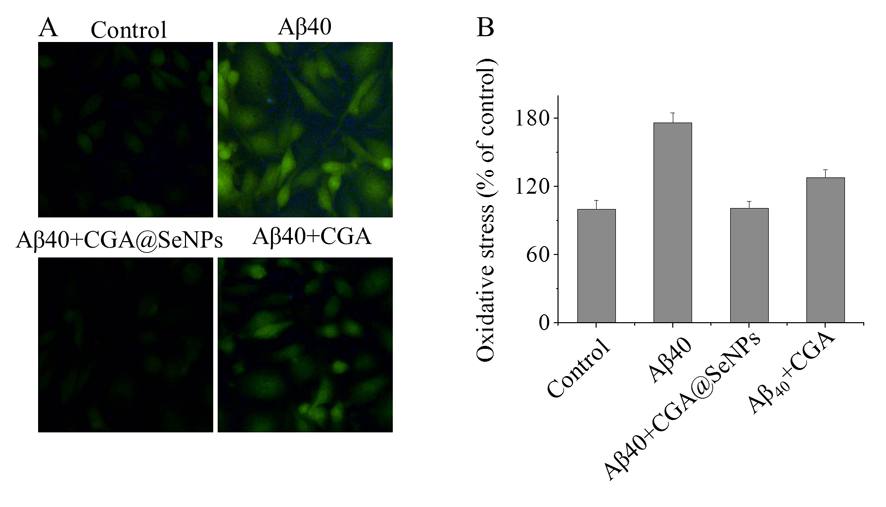


Figure S5. CGA@SeNPs reduced intracellular ROS formation in PC12 cells (A). Quantitative analysis of DCF fluorescence intensity of PC12 cells treated with Aβ40 alone or in the presence of CGA@SeNPs/CGA by a flow cytometer (B). Aβ40=35 μM, CGA@SeNPs/CGA=60 μg/mL.
